# Supplementary material for: Quiz-style online training tool helps to learn birdsong identification and support citizen science
Source: PeerJ. 2023 May 31;11:e15387. doi: 10.7717/peerj.15387 (PMC10239230; doi:10.7717/peerj.15387)
Supplement: Article S1 [file peerj-11-15387-s001.pdf]

事前アンケート

本アンケートは、鳥類音声種判別スキル向上のためのトレーニングについて、その効果の要因を明らかにすることを目的に実施しております。アンケート結果は、本調査に関する研究のデータとして使用させていただきますが、ご回答はすべて統計的に処理し、回答をそのままの形で公表すること、及び、個人が特定できる形で公表することは一切ありません。

また、実験への参加は、対象者自身の自由意思によって決定され、調査の参加に同意した後であっても、いつでも参加を取りやめることができ、そのことによって対象者が不利益を被ることはありません。

メールにて配布いたしました「実験についての説明.docx」、および以下の事項についてご確認のうえ、本実験への参加に同意する場合は、以下のチェックボックスにチェックの上、アンケートにお答えください。本実験を受けることに同意しなくても何ら不利益を受けません。ただし、この同意は、あくまでも自由意思によるものであり、不利益を受けず随時撤回できます。辞退する場合は、******までご連絡ください。

- 「とりトレ」へのログイン情報（URL、ユーザーID 及びパスワード）を第三者に渡さないこと。
- 事前の指示に従って実験に参加すること。
- 実験中に指示に従わない不正行為が確認された場合は謝金を支払わないこと。
- 設定した期間中に実験が終了しなかった場合は、次の日以降の実験に参加できないこと。
- とりトレで紹介されている URL 以外の外部サイトで、野鳥に関する情報を確認しないこと（例：YouTube）。
- とりトレの内容について他の方と相談しない、また、とりトレで得た情報は他者に伝えないこと。

☐ 本実験の参加に同意する ☐ 本実験の参加に同意しない

【野鳥に関する質問】

Q1. 現時点でのご自身の野鳥に対する興味・関心について、どれが当てはまりますか？いずれかの番号をクリックしてください。*必須

1. とても興味・関心がある
2. 興味・関心がある
3. どちらでもない
4. 興味・関心がない
5. まったく興味・関心がない
6. 回答しない

Q2. 現時点でのご自身の野鳥に関する興味・関心について、以下に挙げた詳細の項目ではどれが当てはまりますか？
いずれかの番号をクリックしてください。

- **バードウォッチング（野鳥観察）について***必須

  バードウォッチング（野鳥観察）とは、野鳥調査、調査を目的としない趣味のバードウォッチング（野鳥観察）、他の野外活動（登山・散歩等）に際して副次的に行うバードウォッチング（野鳥観察）等を含みます。

  1. とても興味・関心がある
  2. 興味・関心がある
  3. どちらでもない
  4. 興味・関心がない
  5. まったく興味・関心がない
  6. 回答しない

- **ご自身が野鳥の鳴き声を学習することについて***必須

  1. とても興味・関心がある
  2. 興味・関心がある
  3. どちらでもない
  4. 興味・関心がない
  5. まったく興味・関心がない
  6. 回答しない

Q3. ご自身のバードウォッチング（野鳥観察）の経験レベルについて、どれが当てはまりますか？いずれかの番号をクリックしてください。*必須

1.

経験なし ○ 2.初心者 ○ 3. 中級者 ○ 4. 上級者 ○ 5. 回答しない **Q4. ご自身の鳥の鳴き声による種判別（鳥類の鳴き声から種名を把握すること）の経験レベルについて、どれが当てはまりますか？いずれかの番号をクリックしてください。***必須 ○ 1. 経験なし ○ 2.初心者 ○ 3. 中級者 ○ 4. 上級者 ○ 5. 回答しない

【自然体験等に関する質問】

Q5. ここ１年、自然体験活動を行ったことがありますか。いずれかの番号をクリックしてください。*必須 自然体験活動は、「登山やキャンプ、ハイキング等といった野外活動、又は星空観察や動植物観察といった自然・環境に係る学習活動」のことを指します。

○ 1. はい ○ 2. いいえ ○ 3. 回答しない

Q6. 環境保全活動に参加したことがありますか。いずれかの番号をクリックしてください。*必須 「環境保全活動」は、「社会を構成する個人、家庭、民間団体、事業者、行政等といったあらゆる主体が、環境問題（気候変動対策、循環型社会の形成、生物多様性の保全をはじめとする問題）を自らの問題としてとらえ、自発的に手足を動かして取り組んでいこうとする活動」のことを指します。例えば、公園や樹林・農地・河川・山岳地・海辺等における、草刈り、植林、樹木の剪定、清掃活動、農作業体験、自然教室、動植物の調査、外来生物除去、登山道整備などの活動です。

○ 1. はい ○ 2. いいえ ○ 3. 回答しない

Q7. 今までに鳥（例えばインコ・文鳥など）を飼ったことがありますか？いずれかの番号をクリックしてください。*必須 ○ 1. はい ○ 2. いいえ ○ 3. 回答しない

【この調査に関する質問】

Q8. この調査に協力しようと思われた理由について当てはまるものをクリックしてください。（複数回答可）*必須☐ 1. 鳥の鳴き声を覚えたい ☐ 2. 鳥類のモニタリング調査の発展に協力したい ☐ 3. 効果測定の研究に協力したい ☐ 4. 時間があった

☐ 5. 報酬がほしかった ☐ 6. その他 その他の場合[ ] ☐ 7. 回答しない

回答内容の確認

事後アンケート

最後に、事後アンケートにご協力いただければ幸いです。
本アンケートは、鳥類音声種判別スキル向上のためのトレーニングについて、その効果を明らかにすること、本サイトをよりよいものにすることを目的に実施しております。
アンケート結果は、本調査に関する研究のデータとして使用させていただきますが、ご回答はすべて統計的に処理し、回答をそのままの形で公表すること、及び、個人が特定できる形で公表することは一切ありません。

とりトレ事後アンケート

【野鳥に関する質問】

Q1. ご自身の野鳥に対する興味・関心について、クイズトレーニングを行うことで変化はありましたか？いずれかの番号をクリックしてください。*必須

1. とてもあった　2. あった　3. どちらでもない　4. あまりなかった　5. まったくなかった　6. 回答しない

Q2. ご自身の野鳥に対する興味・関心について、クイズトレーニングを行うことで以下に挙げた詳細の項目では変化がありましたか？いずれかの番号をクリックしてください。*必須

- **バードウォッチング（野鳥観察）について**
  バードウォッチング（野鳥観察）とは、野鳥調査、調査を目的としない趣味のバードウォッチング（野鳥観察）、他の野外活動（登山・散歩等）に際して副次的に行うバードウォッチング（野鳥観察）等を含みます。

  1. とてもあった　2. あった　3. どちらでもない　4.

あまりなかった　5. まったくなかった　6. 回答しない

- **ご自身が野鳥の鳴き声を学習することについて**

　1. とてもあった　2. あった　3. どちらでもない　4. あまりなかった　5. まったくなかった　6. 回答しない

Q3. 現時点でのご自身の野鳥に対する興味・関心について、どれが当てはまりますか？いずれかの番号をクリックしてください。*必須　1. とても興味・関心がある　2. 興味・関心がある　3. どちらでもない　4. 興味・関心がない　5. まったく興味・関心がない　6. 回答しない

Q4. 現時点でのご自身の野鳥に関する興味・関心について、以下に挙げた詳細の項目ではどれが当てはまりますか？
いずれかの番号をクリックしてください。

- **バードウォッチング（野鳥観察）について***必須

　1. とても興味・関心がある　2. 興味・関心がある　3. どちらでもない　4. 興味・関心がない　5. まったく興味・関心がない　6. 回答しない

- **ご自身が野鳥の鳴き声を学習することについて***必須

1. とても興味・関心がある　2. 興味・関心がある　3. どちらでもない　4. 興味・関心がない　5. まったく興味・関心がない　6. 回答しない

Q5. とりトレで出題された 26 種の野鳥のなかで、名前を知っている野鳥はどの程度でしたか？いずれかの番号をクリックしてください。*必須

1. すべて知っていた　2. ほぼ知っていた　3. 半分程度知っていた　4. 少し知っていた　5. まったく知らなかった　6. 回答しない

Q6. とりトレで出題された 26 種の野鳥のなかで、鳴き声を聞いたことがある野鳥はどの程度でしたか？いずれかの番号をクリックしてください。*必須

1. すべて知っていた　2. ほぼ知っていた　3. 半分程度知っていた　4. 少し知っていた　5. まったく知らなかった　6. 回答しない

【とりトレに関する質問】

Q7. とりトレでのトレーニングに満足していますか？いずれかの番号をクリックしてください。*必須

1. 満足　2. やや満足　3. どちらともいえない　4. やや不満　5. 不満　6. 回答しない

Q8. 以下の項目について、どれが当てはまりますか？いずれかの番号をクリックしてください。

- **鳴き声による種同定の技能が向上した***必須

1. とてもそう思う 2. そう思う 3. どちらともいえない 4. そう思わない 5. まったくそう思わない 6. 回答しない

- **とりトレの一連の流れがスムーズにできた***必須

1. とてもそう思う 2. そう思う 3. どちらともいえない 4. そう思わない 5. まったくそう思わない 6. 回答しない

- **とりトレに関する説明スライドがわかりやすかった***必須

1. とてもそう思う 2. そう思う 3. どちらともいえない 4. そう思わない 5. まったくそう思わない 6. 回答しない

- **クイズトレーニングツールは使いやすかった***必須

1. とてもそう思う 2. そう思う 3. どちらともいえない 4. そう思わない 5. まったくそう思わない 6. 回答しない

- **トレーニングは大変だった***必須

1. とてもそう思う 2. そう思う 3. どちらともいえない 4. そう思わない 5. まったくそう思わない 6. 回答しない

- **このプログラムが一般公開されたら家族や親しい知人に紹介したい***必須

1. とてもそう思う 2. そう思う 3. どちらともいえない 4. そう思わない 5. まったくそう思わない 6. 回答しない

- **このプログラムが一般公開されたら、またトレーニングをしたい***必須

1. とてもそう思う 2. そう思う 3. どちらともいえない 4. そう思わない 5. まったくそう思わない 6. 回答しない

- **設定した目標(一週間で 200 問のクイズトレーニングを解くこと)***必須

1. とても多い 2. 多い 3. ちょうどいい 4. 少ない 5. とても少ない 6. 回答しない

Q9. もしよろしければ、感想・改善点等ご自由にお書きください。

回答内容の確認
