# Supplementary material for: Quiz-style online training tool helps to learn birdsong identification and support citizen science
Source: PeerJ. 2023 May 31;11:e15387. doi: 10.7717/peerj.15387 (PMC10239230; doi:10.7717/peerj.15387)
Supplement: Data S1 [file peerj-11-15387-s010.zip › Supplemental Article S12.pdf]

Ogawa translated the approval document.

様式１１

課題番号第 2020－1 号
令和２年８月３日

研 究 倫 理 審 査 結 果 通 知 書

申請者（研究責任者）
伊藤　太一　殿

生命環境系長
松　本　　宏

２０２０年７月２０日付けで申請のあった研究倫理について、審査の結果、下記のとおり判定したので通知します。

記

1　課題名

鳥類音声データの市民参加型種判別に資するオンライン・トレーニングにおける有効性評価

2　判　定

■　承　認
□　条件付承認
□　変更の勧告
□　非承認
□　非該当
□　その他

3　理　由（承認以外の場合）
